# Direct Contraction Force Measurements of Engineered Cardiac Tissue Constructs With Inotropic Drug Exposure

**DOI:** 10.3389/fphar.2022.871569

**Published:** 2022-05-03

**Authors:** Maria Koivisto, Milad Mosallaei, Tarja Toimela, Sampo Tuukkanen, Tuula Heinonen

**Affiliations:** ^1^ FHAIVE (Finnish Hub for Development and Validation of Integrated Approaches), Faculty of Medicine and Health Technology, Tampere University, Tampere, Finland; ^2^ Faculty of Medicine and Health Technology, Tampere University, Tampere, Finland

**Keywords:** force measurement, contraction force, conformal coating, cardiac tissue model, *in vitro* model, human-induced pluripotent stem cell–derived cardiomyocytes, inotropic drug

## Abstract

Contractility is one of the most crucial functions of the heart because it is directly related to the maintenance of blood perfusion throughout the body. Both increase and decrease in contractility may cause fatal consequences. Therefore, drug discovery would benefit greatly from reliable testing of candidate molecule effects on contractility capacity. In this study, we further developed a dual-axis piezoelectric force sensor together with our human cell–based vascularized cardiac tissue constructs for cardiac contraction force measurements. The capability to detect drug-induced inotropic effects was tested with a set of known positive and negative inotropic compounds of isoprenaline, milrinone, omecamtiv mecarbil, propranolol, or verapamil in different concentrations. Both positive and negative inotropic effects were measurable, showing that our cardiac contraction force measurement system including a piezoelectric cantilever sensor and a human cell–based cardiac tissue constructs has the potential to be used for testing of inotropic drug effects.

## Introduction

Drug-induced adverse cardiac effects are one of the main reasons for discontinuations of drug development and post-approval market withdrawals (Lasser et al., 2002; Li et al., 2016). Thus, drug discovery and development would greatly benefit early detection of cardiotoxic reactions. Furthermore, an adequate cardiac tissue model would be very useful for efficacy determination and mechanistic studies. In addition to ethical concerns, extrapolation from animal studies is impeded by species-specific differences in cardiac electrophysiology (Takeda et al., 2018). Inhibition of human Ether-a-go-go–related gene (hERG) channel causing Torsades de Pointes arrythmia can be screened *in vitro*, but this ion channel is not the only vulnerable target of drug-induced adverse reactions (Weaver and Valentin, 2019). The cardiac action potential comprises ion currents from several ion channels and disturbance of any of the channels can potentially be harmful, for example, block and enhancement of sodium channel, enhancement of calcium current, and block of potassium currents also other than hERG can cause arrythmia (Jonsson et al., 2012). Therefore, *in vitro* test systems reliably mimicking the human heart tissue have great potency to improve the probability of success of drug development.

Currently, *in vitro* cardiotoxicity and efficacy testing of drugs lack standardized testing methods for drug-induced inotropic effects on cardiac cells, even though the cardiac contractility directly affects the cardiac output (Guth et al., 2019). Approaches to measure cardiac contraction force *in vitro* include different imaging-based approaches (Rodriguez et al., 2014; Mannhardt et al., 2016) and direct force measurements such as atomic force microscope (Saenz Cogollo et al., 2011), measuring the deflection of an elastic sheet (Linder et al., 2010), and piezoresistive sensor–integrated polydimethylsiloxane (PDMS) cantilever (Kim et al., 2017).

Piezoelectric materials are commonly used in engineering applications such as sensors and actuators due to their mechanical to electrical and electrical to mechanical conversion. This feature is a result of interaction between electrical and mechanical properties of piezoelectric materials (Aabid et al., 2021). The unique mechanical–electrical conversion can also be used in medical devices such as smart sensing applications (Zaszczyńska et al., 2020).

Previously, we have developed a piezoelectric cantilever sensor which can detect low forces, down to sub-µN range, in dual-axis plane directions (Virtanen et al., 2020a). We have successfully measured the contraction force of engineered cardiac tissue constructs previously using the sensor (Virtanen et al., 2020b; Virtanen et al., 2021). The challenge observed with the sensor was that the humidity in the surrounding atmosphere may be absorbed by the surface of the piezo materials and negatively affects the functionality of the materials. Water absorption decreases the mechanical quality and resistivity of the piezo materials. The humidity can also degrade the dielectric strength of the piezoelectric materials (Surbhi and Sukesha, 2020). This is especially a concern with the *in vitro* cell or tissue measurements where an aqueous cultivation medium is present. These issues can be addressed by protection of these materials against humidity, especially in environments with no humidity control. Encapsulation is one approach that is commonly used to coat the piezo materials. Depending on the service condition of the device, different encapsulation materials are used. The demand for using biofriendly encapsulation in medical devices has increased to prevent unwanted interaction of these applications with live organs (Kuppusami and Oskouei, 2015). Parylene is one of the common polymer coating materials used in biomedical applications, which has favorable characteristics including inherent biocompatibility, biostability, and conformal nature of the deposited film (Zeniieh et al., 2013; Kuppusami and Oskouei, 2015). The vacuum-deposited parylene coating can effectively protect the piezo sensors against the incursion of humidity.

Here, we have applied parylene coating to our piezoelectric cantilever sensors and tested its suitability to measure the effects of five inotropic drugs on engineered cardiac tissue contraction force *in vitro*. We could measure both positive and negative inotropic drug effects with the system along with chronotropic responses to the drugs.

## Materials and Methods

### Cell Culture

The use of human adipose stromal cells (hASCs) obtained from surgical operations and the use of human umbilical vein endothelial cells (HUVECs) obtained from scheduled cesarean sections were approved by the Ethics Committee of Pirkanmaa Hospital District (permit numbers R15161 and R15033, respectively). The hASCs and HUVECs were isolated and propagated as previously described (Sarkanen et al., 2012; Toimela et al., 2017). They were maintained in a humidified incubator at +37°C and 5% CO₂, and the medium was refreshed every 2–3 days.

Cardiac tissue models were cultured using a method modified from a previous work (Virtanen et al., 2021). A fibrin hydrogel was prepared in 96-well plates by mixing 50 µL of a solution containing 5.5 mg/ml fibrinogen (Sigma Aldrich, F3879) with 38 μg/ml aprotinin (Sigma Aldrich, A1153) and 50 µL of 2.75 UN/ml thrombin (Sigma Aldrich, T7009). They were incubated for 45 min at +37°C before seeding of hASCs at 20,000 cells/cm^2^ on the fibrin hydrogel. HUVECs were seeded on top of them at 4,000 cells/cm^2^ and 1–4 h later in EBM-2 with EGM-2 SingleQuots supplements (Lonza, CC-3162), as previously described (Toimela et al., 2017). Angiogenesis stimulation was initiated using a serum-free stimulation medium (SFSM) comprising DMEM/F12, 2.56 mM l-glutamine, 0.1 nM 3,3′,5-triiodo-l-thyronine sodium salt, ITS™ Premix: 1.15 μM: 6.65 μg/ml insulin, 6.65 μg/ml transferrin, 6.65 ng/ml selenious acid, 1% bovine serum albumin, 2.8 mM sodium pyruvate, 200 μg/ml ascorbic acid, 0.5 μg/ml heparin, 2 μg/ml hydrocortisone, 10 ng/ml VEGF, and 1 ng/ml FGF-β on the next day, as described earlier (Huttala et al., 2015). After 7 days, the cells were treated with 1 μg/ml mitomycin C (Millipore, 47589). On the next day, hiPSC-derived cardiomyocytes (iCell^2^, Cellular Dynamics, lot. 105451 and 105455) were seeded on top of the vascular-like networks at 312000 cells/cm^2^ in iCell Cardiomyocytes Plating Medium (Cellular Dynamics, M1001). The plating medium was replaced with 1:1 SFSM and iCell Cardiomyocytes Maintenance Medium (Cellular Dynamics, M1003) 4 h later. The cells were maintained in a humidified incubator at +37°C and 5% CO₂, and the medium was refreshed every second day. The whole cell culture process was repeated five times.

### Sensor Preparation

The dual-axis L-shaped force measurement sensors were fabricated and calibrated, as previously described (Virtanen et al., 2021). In brief, two lead zirconate titanate (PZT) piezoelectric sensing elements were cut in desired dimension and soldered together in a position of 90° against each other. One end of the L-shaped structure is attached to a printed circuit board, while the other end is soldered to a cantilever beam.

The functionality and calibration of the fabricated sensors were verified by a texture analyzer Stable Micro Systems TX.XTPlus before the actual measurement on the heart tissues. To carry out this process, 1 Hz sinusoidal displacement amplitude of 100 µm (±50 µm) was subjected to the tip of the piezo sensor for 10 cycles. The loading/unloading was applied by a probe attached to a calibrated load cell of 500 g, which provided a high-resolution force output. While the texture analyzer measured the force required for the displacement, the corresponded sensor output was measured simultaneously. The output data from the texture analyzer and the sensor were then compared together to find the convergence factor in order to calculate the actual construction force of the measured heart tissue constructs.

**FIGURE 1:**
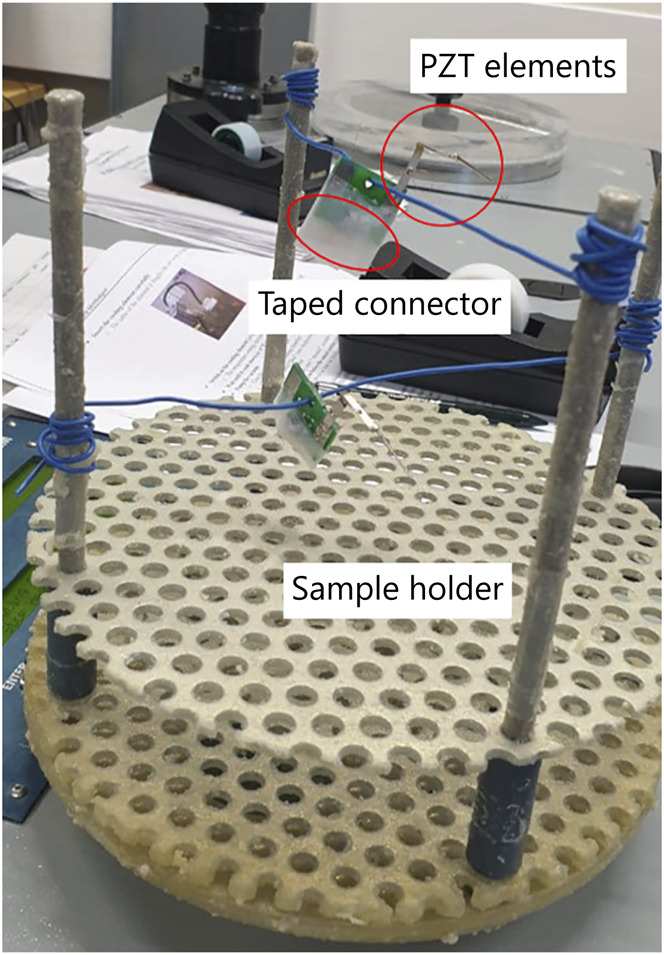
Attachment of the sensor in the sample holder of parylene coating equipment and keeping them in a standing condition with a piece of wire for uniform coating of the polymer in all sides of the sensor during the deposition.

The deposition of parylene was conducted by addition of the polymer in the vacuum chamber using a ParaTech LabTop 3000 Parylene Coater. Before the coating process, the sensors were placed in a sample holder of the parylene coater, and the connectors were taped to prevent the coating materials from getting inside the connectors, as shown in Figure 1. Next, the sample holder was placed inside the vacuum chamber. The procedure for making a 10-µm-thick coating was chosen from the software of the coating machine. In brief, 10 g of a raw powder called dimer (dichloro-p-cyclophane, C_16_H_14_C_l2_) was fed in the heating tube of the coater. The dimer was first heated up in the vacuum atmosphere vaporizing the dimeric gas. Next, the dimeric gas was pyrolyzed to the monomeric form of the dimer, and the gas was deposited on the surface of the sample located in the vacuum chamber which was held at room temperature. At the end of the process, a transparent thin film of parylene was coated on the sample (Kuppusami and Oskouei, 2015).

### Inotropic Drugs

Isoproterenol (ISO; Sigma Aldrich, I5627), milrinone (MIL; Tocris, 1,504), omecamtiv mecarbil (OME; Adooq Biosciences, A11206), verapamil (VER; Tocris, 0654), and propranolol (PRO; Sigma Aldrich, P0884) solutions were prepared freshly before use. Isoproterenol and verapamil were solubilized in distilled water (Gibco, 15230-071) as 10 mM stock. Propranolol and omecamtiv mecarbil were solubilized in DMSO (Sigma, D2650) as 10 mM stock and milrinone as 50 mM stock. They were diluted in L-15 medium (Gibco, 11415-049). During measurements, the test compounds were administered directly to the wells, diluting them to the final concentrations. The amount of DMSO in the final concentrations of omecamtiv mecarbil and propranolol did not exceed 0.003%. For milrinone, the amount of DMSO in the final concentrations was 0.002, 0.02, and 0.2%.

### Force Measurements

**FIGURE 2:**
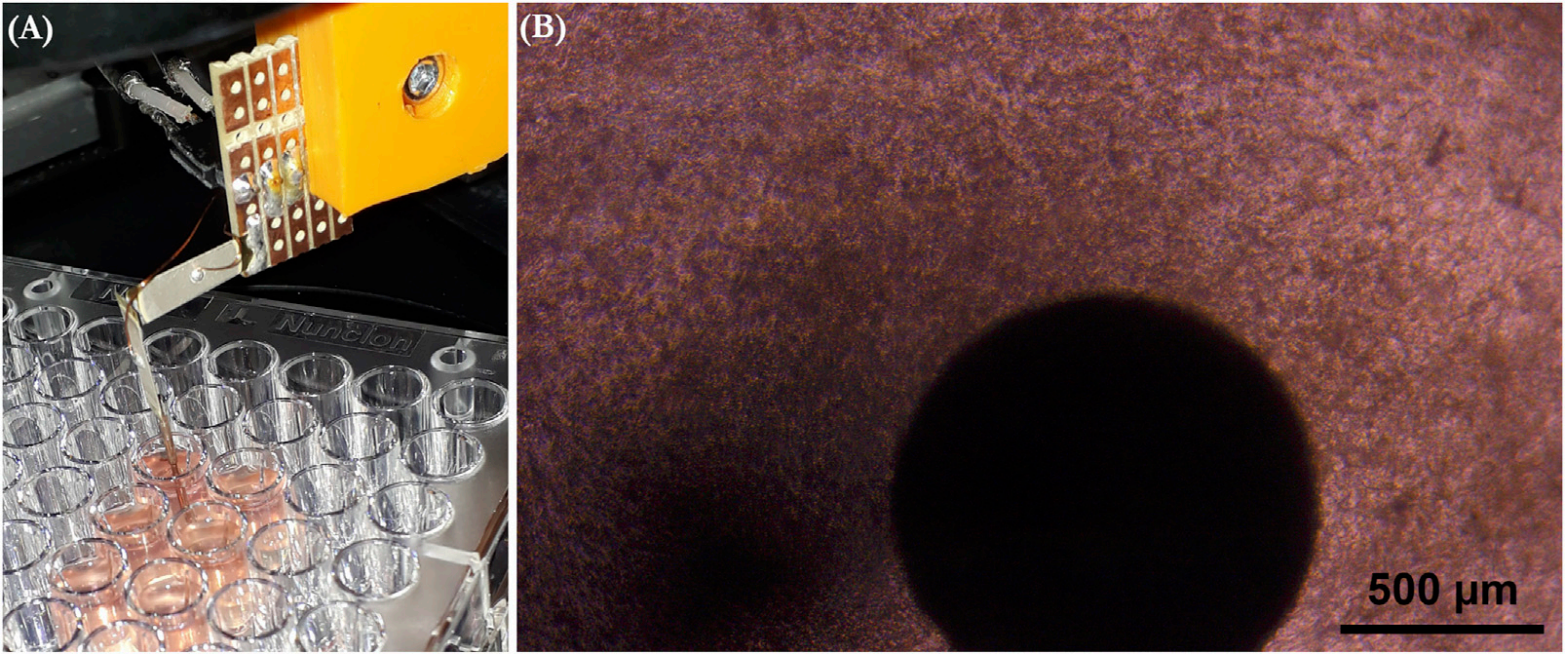
**(A)** Measurement setup where the culture plate is placed on a heater plate under a microscope, and the measurement sensor is brought into contact with a cardiac tissue construct. **(B)** Microscope view of the sensor tip attached onto the cardiac tissue construct.

The force measurement setup and data acquisition platform were similar to those in our previous work (Virtanen et al., 2021). Synchronously beating cardiac tissue constructs were measured 6–9 days after cardiomyocyte seeding. The cell culture plates with beating cardiac tissue constructs were placed on a heater plate set to +37°C on an optical microscope (Zeiss Primovert, Carl Zeiss AG, Oberkochen, Germany), as shown in Figure 2. The cantilever tip was coated by dipping it to 5.5 mg/ml fibrinogen (Sigma Aldrich, F3879) and 2.75 UN/ml thrombin (Sigma Aldrich, T7009) solutions, as previously described (Virtanen et al., 2020b), before contacting the tip to each cardiac tissue construct using a 3-axis linear micromanipulator (Newport Corporation, Irvine, United States). First, a 3-min baseline activity was measured, followed by administration of the first concentration of the test drug from the cumulative concentration series. The activity was measured 10 min after adding each drug concentration. In total, four individual cantilever sensors were used in the measurements.

### Immunocytochemistry

Immunocytochemical staining was performed to confirm the presence of cardiomyocytes and vascular networks in the cardiac tissue constructs. The cells were fixed with 70% ethanol for 20 min on day 9 after cardiomyocyte seeding. The cells were stained with cardiomyocyte-specific monoclonal mouse anti-troponin T antibody (1:100, Invitrogen, MA5-12960, lot UF2730196) and endothelial cell–specific polyclonal rabbit anti–von Willebrand factor (1:100, Dako, A0082, lot 20067357) at +4°C overnight. Secondary antibodies anti-mouse IgG Alexa Fluor 488 (Invitrogen, A21202, lot 2018296) and anti-rabbit IgG Alexa Fluor 594 (Invitrogen, A21207, lot 2066086) were incubated for 45 min at RT. The cells were imaged using an Olympus IX51 inverted fluorescence microscope using a ×10 objective. The images were prepared with Photoshop CC (Adobe).

### Data Analysis

Data were analyzed in Octave software. 2-minute periods of the recordings were included in the analyses, taking 2 min from the end of the baseline measurement and 2 min from 8 to 10 min after administration of each drug concentration in the same cardiac tissue construct. The wells with regular baseline beating were included in the analysis. Data were filtered using a moving average filter with a window size of 2 s. Filtered data were used for peak detection. The peaks were detected separately from both sides by finding the local maxima above an adaptive threshold that was set to include 4–10% of the highest force values in the filtered data. The up and down peak pairs were formed by selecting the closest peaks. Peak-to-peak amplitudes were calculated for the peak pairs from the unfiltered data. Beating frequency was calculated from the peak intervals. For each well, the results from either the x or y channel of the dual-axis sensor were selected based on peak detection. The channel from which more peaks were detected was selected. The same channel was selected from the four consecutive measurements of the same well. The effects of the drugs were normalized for comparison by calculating the percentage change from the baseline activity of each well.

Statistical analyses were performed in IBM SPSS Statistics 27. The nonparametric Kruskal–Wallis test with Bonferroni correction was used for testing the statistical significance of the changes in beat frequency and peak-to-peak forces in different drug concentrations and baseline measurements. The p-values <0.05 were considered statistically significant.

## Results

The beat rate and contraction force values of the baseline measurements together with the percentage change from the cumulative concentrations of the drugs are shown in Table 1. The mean beat rate was 21.2 BPM (SD 7.0) and the mean peak-to-peak contraction force amplitude 331.0 µN (SD 602.2) during the baseline measurements (N = 40 wells).

**TABLE 1:** Mean beating rate and peak-to-peak force amplitudes from the baseline measurements and their percentage changes in the cumulative concentrations of the tested drugs normalized to the baseline measurements.

Drug	Concentration	Mean beating rate	SD	Mean peak-to-peak force	SD	Number of wells
ISO	0 nM	25.87 BPM	6.44	88.95 µN	82.15	6
	10 nM	176.05%	33.79	105.28%	9.84	6
	30 nM	183.86%	32.59	109.56%	10.62	6
	100 nM	193.72%	31.50	115.76%	12.72	6
MIL	0 µM	20.76 BPM	5.95	465.44 µN	622.6	9
	1 µM	108.13%	16.73	103.06%	9.91	9
	10 µM	118.06%	19.11	112.15%	12.38	9
	100 µM	232.67%	50.05	103.51%	14.19	9
OME	0 nM	22.22 BPM	6.77	37.45 µN	16.90	10
	30 nM	96.31%	12.49	98.45%	5.819	10
	100 nM	94.76%	17.67	95.53%	13.20	10
	300 nM	94.51%	19.36	95.81%	12.23	10
PRO	0 nM	21.47 BPM	4.856	673.27 µN	822.83	9
	30 nM	98.62%	14.43	97.18%	9.39	9
	100 nM	90.73%	16.24	94.62%	10.79	9
	300 nM	63.16%	26.93	93.63%	9.03	9
VER	0 nM	15.10 BPM	7.77	347.28 µN	645.90	6
	10 nM	116.01%	31.83	99.53%	12.20	6
	30 nM	115.62%	27.07	93.90%	15.86	6
	100 nM	157.02%	25.54	85.71%	8.26	4

**FIGURE 3:**
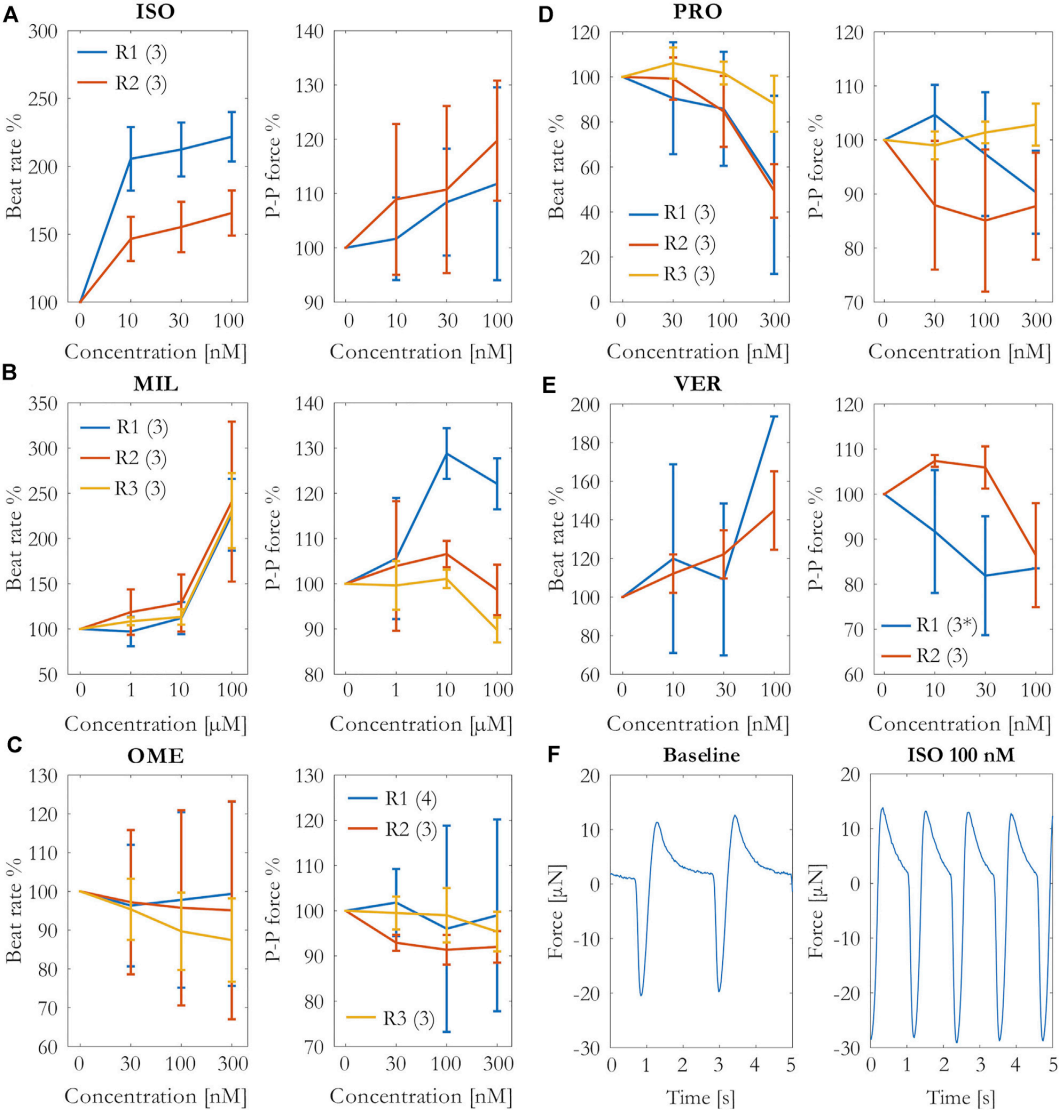
Percentage change of the beating rate and force in the cumulative concentrations of **(A)** isoprenaline (ISO), **(B)** milrinone (MIL), **(C)** omecamtiv mecarbil (OME), **(D)** propranolol (PRO), and **(E)** verapamil (VER) exposures. Mean and SD of the beating rate and peak-to-peak amplitudes from each repeat R1–R3 (number of measurements per repeat). ^*^N = 1 at 100 nM. **(F)** Beating from an example well during baseline and 100 nM isoprenaline force measurements.

The effects of the cumulative concentrations of the tested drugs on the beat rate and contraction force are shown in Figure 3 as percentage change from the baseline measurements. As expected, the positive chrono- and inotrope isoprenaline increased both beating rate and contraction force. However, the variation in the force results was quite high, and only the increase in the beating rate was statistically significant (*p* = 0.016 at 30 nM, *p* = 0.005 at 100 nM, N = 6).

The isoprenaline-induced change in the contraction force and beating rate is clearly visible in the example signal in Figure 3. The second positive inotrope, milrinone, increased the beating rate with statistical significance (p < 0.001 at 100 μM, N = 9). The effects of milrinone on the contraction force were incoherent among the repeats. While the drug increased the force during the repeat R1, it failed to increase it during the repeats R2 and R3.

Omecamtiv mecarbil induced nonuniform effects on the beating rate among the different wells, in some wells the effect being positive and in others negative chronotropy, causing variation to the results especially in repeats R1 and R2. In repeat R3, the trend was mainly negative chronotropy. Likewise, the results on the contraction force differed among the parallel wells in the repeat R1 from slight increase to slight decrease in force. Overall, the drug did not have an apparent effect on the contraction force. We also tested higher concentrations (600 nM and 900 nM) of omecamtiv mecarbil without a clearer effect on the force (data not shown).

Propranolol caused a statistically significant decrease in beating rate (*p* = 0.003 at 300 nM, N = 9), but the effect in contraction force was not clear. The contraction force seemed to decrease slightly in repeats R1 at the highest concentration and R2, but it did not change in repeat R3 in which also the decrease in beating rate was the smallest.

Verapamil increased the beating rate in most of the wells but decreased it in one well. Moreover, the beating rate ceased in two wells at 100 nM in repeat R1. Apart from the slight increase at 10 and 30 nM in repeat R2, the contraction force was decreased by verapamil. However, the decrease was not statistically significant.

**FIGURE 4:**
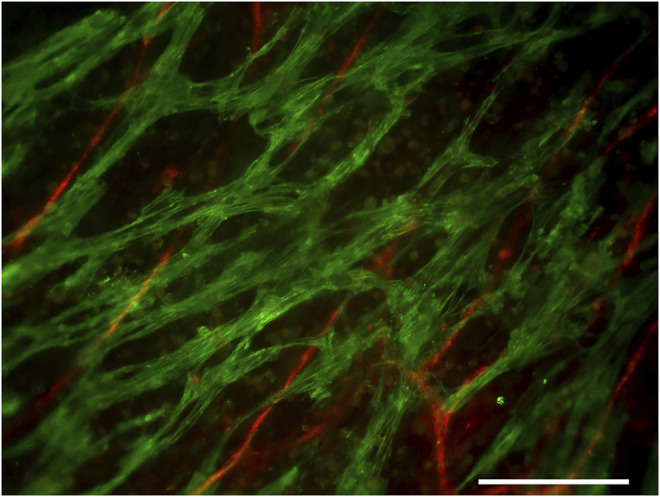
Cardiac troponin T (green)–positive cardiomyocytes and the von Willebrand factor (red)–positive vascular network. Scale bar: 200 µm.

Immunocytochemical staining confirmed the presence of cardiac troponin T–positive cardiomyocytes and the von Willebrand factor–positive vascular network in the cardiac tissue constructs (Figure 4). The cardiomyocytes were connected to each other and arranged into a network. The connectiveness of the cardiomyocytes was also indicated by the synchronous beating of the whole construct in each well during the force measurements.

## Discussion

Isoprenaline is a *β*-adrenergic receptor agonist and a positive inotrope. The positive inotropic effect has been previously reported in cardiac microtissues consisting of hiPSC-CM and fibroblasts (Huebsch et al., 2016) and in hiPSC-derived cardiomyocytes, for example, 51% increase in force at 10 nM (Saleem et al., 2020) and 41% increase in force at 100 nM (Mannhardt et al., 2016). Positive chrono- and inotropy at 1 µM has been reported in cardiac constructs consisting of hiPSC-CM, fibroblasts, and endothelial cells (Arai et al., 2020). Our results are well in line with those of the previous studies, although the changes were not statistically significant. The higher relative beating rates in repeat R1 can at least partly be explained by their slower baseline beating rate than those of repeat R2, which enables higher relative increases in beating rates. The chrono- and inotropic effects of isoprenaline were measurable and repeatable in our system.

Milrinone is primarily a phosphodiesterase type-3 (PDE3) inhibitor that increases intracellular calcium levels and contraction force in human heart. The drug has also been reported to lack the effect on hiPSC-derived cardiomyocytes *in vitro* (Mannhardt et al., 2017; Saleem et al., 2020), which is similar to the second and third repeats in our study. Saleem et al. (2020) concluded that the lack of inotropic effect of milrinone in their study was most likely due to the lower expression of PDE3A and PDE4A than that of human heart samples and the predominance of PDE4 isoform over PDE3 in their engineered heart tissue, which could be linked to the immaturity of the cardiomyocytes. Sube and Ertel (2017) reported higher susceptibility to PDE4 inhibition than that to PDE3 in their hiPSC-CM cultures too. In contrast, Feric et al. (2019) obtained a maximal 4.3 ± 1.2-fold increase in the contraction force at 100 µM milrinone with an EC_50_ of 1.6 μM in their engineered cardiac tissues containing cardiomyocytes and fibroblasts, and which had been electrically stimulated during the culturing to improve maturation. In the study of Ravenscroft et al. (2016), milrinone increased contraction force with EC_50_ = 83.68 µM in cardiac triculture microtissues containing endothelial cells and fibroblasts but did not have an effect on bare cardiomyocyte microtissues. Our cardiac tissue model contains several relevant cell types including endothelial cells and myofibroblasts that promote the cardiomyocyte maturation, which is further compared to monoculture of cardiomyocytes (Koivisto, M, Tolvanen, T.A, Toimela, T, Miinalainen, I, Kiviaho, A, Kesseli, J, et al. (2022). “Functional human cell–based vascularized cardiac tissue model for biomedical research and testing” [manuscript submitted for publication]). The improved maturation may enable the positive inotropic effect of milrinone in our cardiac constructs, even though it was not observed in all repeats.

Despite differences in the force results among the milrinone repeats in our study, the positive chronotropic effect was evident at the highest concentration in all repeats. The positive chronotropic effect of milrinone has been previously reported at concentrations ≥3 µM in hiPSC-CM cultures (Sube and Ertel, 2017). However, the positive chronotropic effect could also be caused by the vehicle 0.2% DMSO at that concentration in our study because DMSO has been reported to increase the beating rate of hiPSC-CMs (Jonsson et al., 2011). The effect of DMSO could also explain the decrease of contraction force in all repeats at the highest milrinone concentration.

Omecamtiv mecarbil is a selective cardiac myosin activator that produces positive inotropy without affecting intracellular calcium levels (Planelles-Herrero et al., 2017). Positive inotropic effect has also been reported *in vitro,* for example, Feric et al. (2019) measured a maximal 2.6 ± 0.3-fold increase in contractile force in their engineered human cardiac tissue at 10 µM omecamtiv mecarbil with an EC_50_ of 370 nM. However, varying inotropic effects have also been reported. Measuring from single-cell hiPSC-CMs, Ballan et al. (2020) reported 11% increase in force at 100 nM but higher doses, 1 and 10 µM, suppressed contractility. Ribeiro et al. (2017), also measuring single-cell hiPSC-CMs, reported positive inotropy immediately after administration of 100 nM omecamtiv mecarbil. However, 5 min after the exposure, they measured negative inotropy at 10 and 100 nM, possibly due to the myofibril damage that they detected. In our study, we did not obtain a clear result in force amplitude from omecamtiv mecarbil, even though some of the wells showed positive or negative inotropic responses especially in repeat R1. Ribeiro et al. (2017) obtained positive chronotropic effects of omecamtiv mecarbil. In contrast, negative chronotropy of omecamtiv mecarbil has been reported in clinical trials (Teerlink et al., 2020). In our study, even though the beating rate seemed to decrease in repeat R3, the results from repeats R2 and R3 were inconclusive with high variation among the parallel wells, some wells showing increase and some decrease or no change in the beating rate.

Propranolol, by blocking *β*-adrenergic receptors, results in negative chrono- and inotropy in human heart. In the absence of adrenergic stimulation, propranolol would not be expected to cause negative inotropic effects. However, propranolol has also been reported to block cardiac NaV1.5 sodium channels (Wang et al., 2010). In our study, only the negative chronotropic effect was clear, which might result from propranolol blocking the sodium channels. The negative chronotropic and inotropic effects have been previously measured *in vitro*, for example, in hiPSC-CM constructs (Morimoto et al., 2016) and cardiac constructs consisting of hiPSC-CM, fibroblasts, and endothelial cells (Arai et al., 2020).

Verapamil is an L-type calcium channel blocker and a negative inotrope. Negative inotropy of verapamil has been reported in previous *in vitro* studies, for example, in engineered heart tissue (Mannhardt et al., 2016) and hiPSC-CMs (Gossmann et al., 2016). In our study, apart from the slight increase in force at the lowest concentrations in repeat R2, the overall trend was negative inotropy as expected. Verapamil is clinically used for slowing heart rate. However, in our study, the effect was the opposite in most of the wells. Positive chronotropy of verapamil has been previously reported in dogs (Nakaya et al., 1983). Few of the wells in our study showed decrease in the beating rate and cessation of beating at 100 nM. The beating rate in these wells was already low, that is, <10 BPM during the baseline measurements.

Overall, the beating rate of the cardiac constructs during the baseline measurements was constant among parallel wells and comparable to that of our previous force measurement study where the beating rates were in range 19–38 BPM (Virtanen et al., 2021). Variation in the beating rate can originate from the cell culture batches at different time in culture depending on the measurement days 6–9 days after cardiomyocyte seeding.

Variation in the absolute contraction force values between different measurements results from differences between the sensor elements and especially differences in calibrated amplification of the measurement channels. Although the cardiac tissues were prepared the same way for each repeat, individual cardiac tissue constructs were different in beating strength and location of the strongest beating. The sensor tip was connected with the strongest contraction point, but the direction of cardiac contraction relative to the chosen measurement channel caused variation in the absolute force values among the parallel wells. As the contact point between the cardiac tissue and sensor tip influenced the measured force amplitude, it was critical to maintain the contact point intact during the consecutive measurements of the same well to enable comparison. Because the drug administration was performed manually by pipetting the amount of the drug into the wells, it was possible that the cantilever was touched with the pipette tip, causing disturbance in the contact point between the sensor tip and cardiac tissue construct. Special attention was paid on this and when noticed, the results from the well were omitted from the analyses because the possible difference in force might not be due to the drug effect but the effect of the changed contact point. However, if it remained unnoticed, it could distort the results.

Although complex cellular models best resemble the *in vivo* situation, they are challenging to standardize. Moreover, even advanced models cannot fully represent the complexity of the *in vivo* situation. Our cardiac tissue model does not have connections with nerve stimulation and does not recapitulate the structure of the heart. Moreover, the maturity of the hiPSC-derived cardiomyocytes does not reach the level of adult human cardiomyocytes with the current techniques. Other limitations of this study include inconsistent and low number of measured wells for some of the tested drugs and high variation in the force results among parallels and among different testing times for some of the drugs. The force measurement technique used in our study is currently time-consuming and laborious because only one well can be measured at a time. Moreover, the selection of the measured wells and measurement locations in these wells is manual and prefers cultures with strong and regular beating.

As a conclusion, our cardiac contraction force measurement system including a piezoelectric cantilever sensor and a human cell–based cardiac tissue constructs has potential to be used for testing of inotropic drug effects. Even though the variation of the force results among parallel wells or repeats was relatively high for some of the tested drugs, it was possible to detect both positive and negative inotropic effects in this system.

## Data Availability

The raw data supporting the conclusion of this article will be made available by the authors, without undue reservation.
